# Quantitative Proteome and Transcriptome Dynamics Analysis Reveals Iron Deficiency Response Networks and Signature in Neuronal Cells

**DOI:** 10.3390/molecules27020484

**Published:** 2022-01-13

**Authors:** Luke Erber, Shirelle Liu, Yao Gong, Phu Tran, Yue Chen

**Affiliations:** 1 Department of Biochemistry, Molecular Biology and Biophysics, University of Minnesota at Twin Cities, Minneapolis, MN 55455, USA; lerber@umn.edu (L.E.); gong0062@umn.edu (Y.G.); 2 Department of Pediatrics, University of Minnesota at Twin Cities, Minneapolis, MN 55455, USA; liu00459@umn.edu

**Keywords:** oxygen sensing, hypoxia, iron deficiency, quantitative proteomics, transcriptome analysis, neuronal cells, hippocampus

## Abstract

Iron and oxygen deficiencies are common features in pathophysiological conditions, such as ischemia, neurological diseases, and cancer. Cellular adaptive responses to such deficiencies include repression of mitochondrial respiration, promotion of angiogenesis, and cell cycle control. We applied a systematic proteomics analysis to determine the global proteomic changes caused by acute hypoxia and chronic and acute iron deficiency (ID) in hippocampal neuronal cells. Our analysis identified over 8600 proteins, revealing similar and differential effects of each treatment on activation and inhibition of pathways regulating neuronal development. In addition, comparative analysis of ID-induced proteomics changes in cultured cells and transcriptomic changes in the rat hippocampus identified common altered pathways, indicating specific neuronal effects. Transcription factor enrichment and correlation analysis identified key transcription factors that were activated in both cultured cells and tissue by iron deficiency, including those implicated in iron regulation, such as HIF1, NFY, and NRF1. We further identified MEF2 as a novel transcription factor whose activity was induced by ID in both HT22 proteome and rat hippocampal transcriptome, thus linking iron deficiency to MEF2-dependent cellular signaling pathways in neuronal development. Taken together, our study results identified diverse signaling networks that were differentially regulated by hypoxia and ID in neuronal cells.

## 1. Introduction

Microenvironment sensing is critical in maintaining normal physiology and developmental activity in mammalian cells and tissues [1,2,3,4,5]. Rapid adaptation to microenvironmental changes involves complex and diverse signaling mechanisms that serve as “sensors” to the extracellular cues [5,6,7]. Oxygen is one of the key factors in the cellular microenvironment that strongly affects energy homeostasis. Low oxygen availability (hypoxia) inhibits the oxidative phosphorylation-mediated energy production and promotes neuronal cell death, contributing to tissue injury in ischemic brain [8]. Studies in the past two decades have established hypoxia-inducible factors (HIF1-a) as a sensor and master regulator of cellular hypoxia-responsive pathways [9,10,11]. Under normoxia, hydroxylation of HIF1-a by prolyl hydroxylases PHDs (or EGLNs) promotes its interaction with ubiquitin E3 ligase von Hippel–Lindau (pVHL) and leads to the poly-ubiquitination and subsequent rapid degradation of HIF1-a [12,13,14]. Hypoxia condition inhibits the hydroxylation and subsequent rapid degradation of HIF1-a, which leads to its nuclear enrichment and induction of hypoxia-responsive genes. Recent system-wide analyses using transcriptome profiling and protein quantification have further expanded this body of work by uncovering substantial hypoxia-induced changes in gene expression and protein abundances critical for neuroplasticity in neuronal cells and brain tissues [15,16,17].

Iron is a key co-factor for many oxygen-related enzymes and cellular processes, playing essential roles in neuronal development [1,2,3,18,19]. It is required for the enzymatic activity of dioxygenases, including prolyl hydroxylases, that hydroxylate HIF1-a and demethylases that removes epigenetic methyl marks from histones [20]. Insufficient uptake of iron from the cellular microenvironment leads to iron deficiency (ID) and abnormal neuronal cell development [1,2]. As with hypoxia, ID also leads to the inhibition of HIF1-a hydroxylation and degradation, thereby upregulating HIF1-a dependent gene expression [4,21]. However, the effects of cellular ID on signaling networks regulating cellular physiology and homeostasis have not been fully determined [4,22]. System-wide analysis of ID in mammalian cells and tissues has been largely limited to transcriptional profiling of gene expression that revealed changes in expression of genes regulating growth arrest and DNA damage response in developing neurons or neuroblastoma cell lines [23,24,25,26].

In the present study, we applied a global proteomic screening in combination with stable isotopic labeling of amino acids in cell culture (SILAC) [27] to identify early changes in cellular signaling pathways caused by hypoxia and ID in hippocampal-derived neuronal cell line. Our deep proteomic analysis provided new insights into the hypoxia-induced dynamics of metabolic pathways and epigenetic regulations and identified diverse signaling networks that were differentially regulated by hypoxia and ID in neuronal cells.

## 2. Results

### 2.1. Experimental Strategy for the Quantification of the Iron and Oxygen Starvation-Dependent Proteome

**Figure 1:**
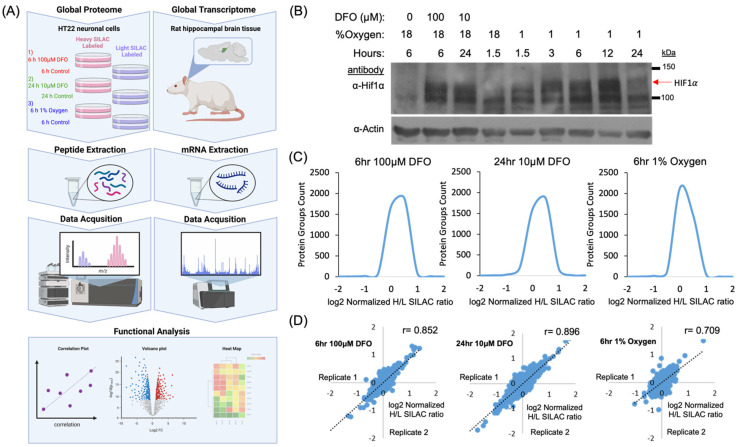
Experimental design for comprehensive analysis of the global proteome. (**A**) Outline of experimental workflow for comprehensive analysis of the global proteome and transcriptome. To study the global proteome, stable isotopic labeling of amino acids in cell culture (SILAC)-labeled HT22 cells were treated under hypoxic and iron deficient conditions. Peptides were extracted and characterized by mass spectrometry. To study the global transcriptome, hippocampal tissue from iron-deficient rat (P15) were extracted. mRNA was purified and characterized by a microarray study reported previously [36]. Both datasets were processed using functional analysis tools. (**B**) Western blotting of HT22 cells treated with either 100μM DFO for 6 h, 10M DFO for 24 h, or 1% oxygen for 1.5, 3, 6, 12, and 24 h and their respective non-treated controls. Red arrow indicates HIF1A. (**C**) Protein groups count distributed by normalized H/L SILAC ratio. (**D**) Plots showing biological replicate sample correlation after quantification of protein SILAC ratios.

To reveal the proteome dynamics following hypoxia and iron starvation in neurons, we utilized the mouse hippocampal HT22 cells for quantitative proteomic analysis (Figure 1A) [28]. To optimize cellular response to hypoxia, we monitored the HIF1-a abundance using Western blotting. Our data showed that HIF1-a level increased after 1.5 h and peaked at 12 h of hypoxic treatment (Figure 1B). Desferoxamine (DFO) is a well-characterized, cell-permeable iron chelator. Acute and chronic ID have been successfully induced by DFO treatment for 6 h at 100 µM concentration [21,29,30,31] or 24 h at 10 µM concentration [18,21,32,33,34]. Six hours of hypoxia treatment produced a similar increase in HIF1-a abundance comparing both acute and chronic ID; therefore, this was chosen as the treatment condition for hypoxia in the global quantitative analysis.

HT22 cells were cultured in SILAC-heavy (containing Lys^8^, Arg^10^) or -light (containing Lys^0^, Arg^0^) media for more than six generations (Figure 1A). For the oxygen starvation treatment, heavy labeled HT22 cells were incubated at 37 °C for 6 h in a hypoxia chamber containing 1% O_2_, 94% N_2_, 5% CO_2_ while the light-labeled HT22 cells were incubated at 37 °C for 6 h under the normoxia condition. To induce ID, two sets of HT22 cells were treated with DFO for 6 h (100 µM, acute ID) or 24 h (10 µM, chronic ID), respectively. Proteins from SILAC “light” and “heavy” cells were equally mixed followed by trypsin digestion. The tryptic peptides were further fractionated using basic high pH reverse-phase offline HPLC to increase the proteome coverage prior to the analysis on nano-HPLC Orbitrap Fusion mass spectrometer [35].

### 2.2. Dynamics of Cellular Proteome in Response to Hypoxia and Iron Deficiency

Our systematic analysis identified 8697 proteins, which annotated 4805 protein groups, at a false discovery rate (FDR) of 1% from two biological replicates (Table S1). Quantitative analysis showed greater than 93% of proteins exhibited no more than a 2-fold change in response to these treatment conditions. These observations suggest that acute hypoxia or iron deficiency caused low–moderate effects on global protein abundance (Figure 1C).

To assess the reliability of our quantitative analysis, we determined the correlation between biological replicates. The correlation of Log2 SILAC quantification ratios between the replicates indicated excellent reproducibility of our quantification data (Figure 1D). Our Western blotting analysis showed that the 6 h hypoxia treatment produced a significant increase in HIF1-a abundance in HT22 cells. To determine whether the increased HIF1-a abundance was sufficient to activate known HIF1-a-mediated hypoxia response pathways, we examined the protein abundance of known HIF1-a targets. Among 40 HIF1-a targets that were identified by LCMS in hypoxic samples, only 2 protein targets showed more than 50% increase in protein abundance, namely Hydroxy-methylglutaryl-CoA lyase (HMGCL) (ratio 1.89) and BCL2/adenovirus E1B 19 kDa protein-interacting protein 3 (BNIP3) (ratio 2.00) (Table S2). These data suggest that our 6-h hypoxia treatment of HT22 cells represents an early hypoxia response.

### 2.3. Dynamics of Biological Pathways Induced by Acute Hypoxia

**Figure 2:**
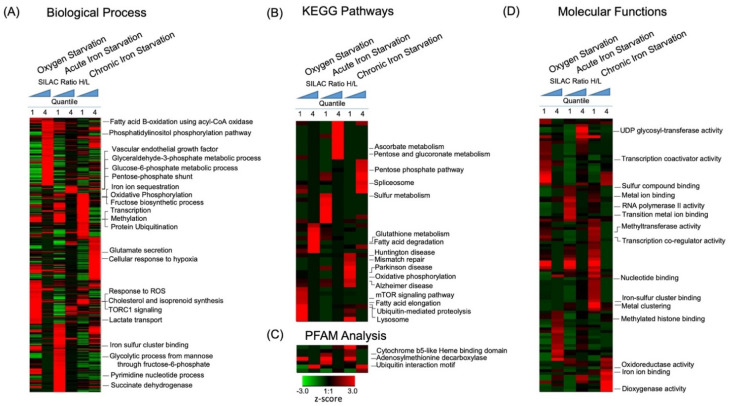
Enrichment and clustering analysis of the oxygen and iron deficiency proteome datasets based on Gene Ontology annotations. Gene Ontology annotation classified genes based on four categories: (**A**) Biological Process, (**B**) KEGG Pathways, (**C**) PFAM analysis, and (**D**) molecular functions. In each category, SILAC quantification ratios of all proteins were divided into four quantiles based on the normalized heavy/light Log2 SILAC ratios (less than −1, −1 to 0, 0 to 1, more than 1). An enrichment test was performed using Benjamini–Hochberg adjustment. The P-values were transformed into z-scores before hierarchical clustering analysis.

To functionally annotate the dynamics of cellular pathways altered by oxygen and iron depletion, we divided the global proteome dataset into four quantiles based on the normalized SILAC-heavy/light (treatment/control) Log2 ratios (<−1, −1 to 0, 0 to +1, >+1). Enrichment analysis was performed separately for each quantile using Gene Ontology (GO) [37], KEGG pathway [38], and PFAM domain databases [39]. Overrepresented annotations were clustered using hierarchical clustering for comparative analysis (Figure 2 and Table 1, Table S3).

#### 2.3.1. Metabolic Processes

**Figure 3:**
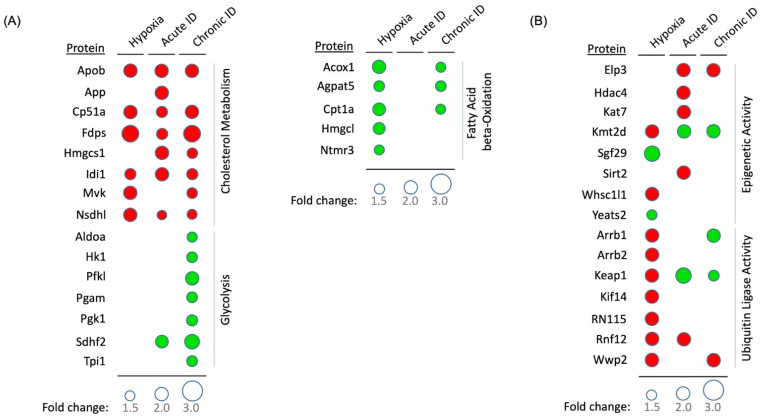
Representative protein abundance altered by hypoxia and iron deficiency. (**A**) Bubble heat map showing proteins having metabolic activity significantly changed under all treatments. Bubble size indicates level of fold change in protein quantitation with SILAC ratio <0.66 (green) and >1.5 (red). (**B**) Bubble heat map showing proteins having epigenetic and ubiquitination activity significantly changed under all treatments. Bubble size indicates level of fold change in phosphorylation site with SILAC ratio <0.66 (green) and >1.5 (red).

Oxygen availability strongly affects the activity of the electron transport chain and ATP production in mitochondria. However, we did not observe significant changes in protein abundance in the oxidative phosphorylation pathway following the acute hypoxia treatment; instead, our data showed significant changes in the fatty acid metabolism pathway. Under acute hypoxia, proteins in fatty acid beta oxidation and degradation pathways were significantly upregulated (Peroxisomal acyl-coenzyme A oxidase 1 (Acox1) and SILAC ratio of 2.0), whereas proteins in fatty acid elongation and biosynthesis pathways (Palmitoyl-protein thioesterase 1 (PPT1)—SILAC ratio of 0.5), as well as cholesterol and isoprenoid metabolic processes, were downregulated (Figure 2A,B and Figure 3A, Table S3). Since fatty acid degradation and biosynthesis processes occur in different cellular compartments (mitochondria and cytosol, respectively), these data suggested a possible compensatory regulation as an immediate neuronal response to hypoxia.

#### 2.3.2. Protein Synthesis, Folding, and Survival Pathways

Our data also showed that acute hypoxia downregulated proteins involved in transcription, protein synthesis, and folding processes (Figure 2A, Table S3). These observations were expected given the significant decrease in ATP production under hypoxia. On the other hand, hypoxia upregulated pathways regulate phosphatidylinositol phosphorylation, apoptosis survival, glutathione metabolism, and cellular endocytosis (Figure 2B, Table S3).

#### 2.3.3. Ubiquitination and Protein Degradation Pathways

The ubiquitination proteasome system plays an important role in regulating protein homeostasis [40]. Hypoxia reduced enrichment of proteins regulating ubiquitination processes, including enzymes possessing specific K29, K6, K11, and K27-linkage ubiquitination (Figure 2B and Figure 3B, Table S3), but enriched proteins in the endoplasmic reticulum (ER)-associated protein degradation pathway (ERAD), consistent with previous findings of hypoxia-induced ER stress [41] (Figure 2A, Table S3).

#### 2.3.4. Regulation of Epigenetic Pathways

Our analysis revealed that acute hypoxia reduced the abundance of proteins with methyltransferase activity, including Histone-lysine N-methyltransferase NSD3 (WHSC1L1–SILAC ratio of 0.5) and Histone-lysine N-methyltransferase 2D (KMT2D–SILAC ratio of 0.5) (Figure 2A,D and Figure 3B, Table S3). In contrast, hypoxia increased the abundance of histone acetyltransferase complex including the YEATS domain-containing protein 2 (YEATS2–SILAC ratio of 1.5) and SAGA-associated factor 29 homolog (SGF29–SILAC ratio of 2.3). These data indicated changes in epigenetic regulation mediated by oxygen-dependent enzymatic activity.

#### 2.3.5. Nutrient-Dependent Cellular Signaling

A central regulator of protein synthesis is the mammalian target of rapamycin mTOR [42]. In agreement with previous findings, hypoxia reduced enrichment of proteins regulating mTOR complex 1 (TORC1, Tuberin/Tsc2–SILAC ratio of 0.5) signaling, protein kinase B-mediated insulin signaling (Eukaryotic translation initiation factor 4E type 2 (EIF4E2)–SILAC ratio of 0.5) [43,44], as well as lysosomal activity and autophagy (VPS13A–SILAC ratio of 0.3, MAP1LC3B–SILAC ratio of 0.6, ATG4B–SILAC ratio of 0.5, and WDR45–SILAC ratio of 0.5) (Figure 2A,B, Table S3).

### 2.4. Identification of Specific Iron-Dependent Cellular Pathways in Neuronal Cells

Comparative analysis of iron and oxygen-dependent proteome dynamics revealed a large number of cellular pathways induced specifically by ID, including metal ion homeostasis, cellular metabolism, and signaling pathways (Figure 2, Table S3). Interestingly, acute and chronic ID showed differential activation or inhibition of cellular pathways, despite the fact that both treatments activated similar HIF1-a levels. Analysis of known HIF1-a targets showed that while chronic ID led to a greater than 50% enrichment of at least 11 known HIF1-a targets, acute ID did not (Table S2).

#### 2.4.1. Metal ion Binding Proteins and Processes

Both acute and chronic ID reduced levels of proteins regulating metal ion homeostasis and related biological processes (Figure 2A, Table S3). These included iron ion sequestration, ferric, and ferrous iron incorporation into metallo–sulfur clusters (ferritin-heavy chain (FTH1)–SILAC ratios of 0.29 and 0.45; ferritin (FTL1)–SILAC ratios of 0.32 and 0.44 for acute and chronic ID, respectively). Analysis of molecular functions and PFAM domains showed that proteins with Fe ion binding and iron–sulfur cluster binding, including those with cytochrome b5-like heme-binding motif, were significantly reduced. These observations are consistent with prior findings, suggesting that loss of iron increased the instability of iron-binding proteins and iron-containing protein complexes (Figure 2D, Table S3) [45,46].

#### 2.4.2. Effects of Iron Deficiency on Regulation of Cellular Metabolism

**Figure 4:**
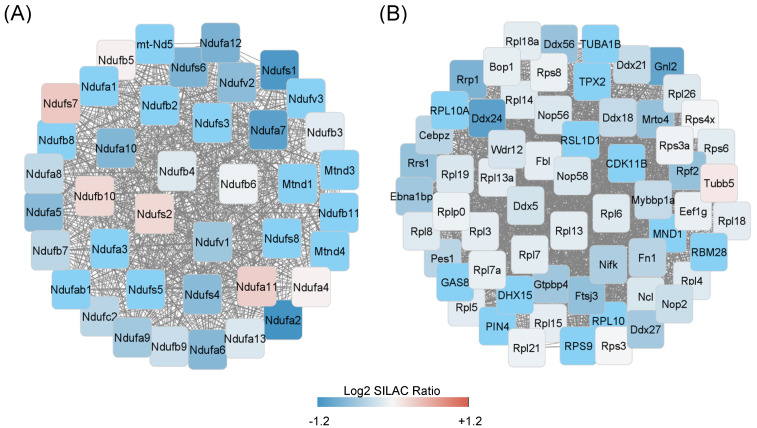
A schematic representation of protein complex interaction networks. Chronic iron deficiency treatment of HT22 cells significantly downregulated mitochondrial respiration chain complex I (**A**) and Parvulin-associated pre-RNP complex (**B**). The node color represented the log2 SILAC ratio of treatment (heavy labeling) vs. control (light labeling).

As with hypoxia, ID significantly lowered enrichment of proteins regulating cholesterol and isoprenoid metabolic processes (Figure 2A, Table S3). Chronic ID upregulated the abundance of proteins involved in the hypoxia response pathways and reduced mitochondria respiratory chain complex in the oxidative phosphorylation pathway (Figure 2A, Table S3). Both acute and chronic ID induced enrichment of proteins in the glycolysis-related pathways including pentose–phosphate shunt, glyceraldehyde-3-phosphate, and glucose 6-phosphate metabolic processes (Figure 2A and Figure 3A, Table S3). Unlike hypoxia, acute ID had little effect while chronic ID moderately increased the abundance of proteins in the fatty acid degradation processes (Figure 2B and Figure 3A, Table S3). CORUM complex enrichment analysis of proteins with greater than 50% reduction by chronic ID revealed significantly reduced abundance of the mitochondria electron transport chain complex I (*p* < 1.2 × 10^−7^, Figure 4A) and Parvulin-associated pre-RNP complex (*p* < 7.9 × 10^−7^, Figure 4B). These findings suggested that chronic ID strongly suppressed mitochondria-mediated oxidative phosphorylation activity and ribosome-mediated protein synthesis in neuronal cells.

#### 2.4.3. Iron-Dependent Regulation of Neuronal Signaling

Both acute and chronic ID caused a significant upregulation of proteins in the vascular endothelial growth factor (VEGF) receptor signaling pathway, which was not upregulated by the acute hypoxia treatment (Figure 2A, Table S3). In addition, chronic but not acute ID significantly upregulated proteins involved in glutamate release, a major neuronal excitatory pathway (Figure 2A, Table S3). Analysis of KEGG pathways showed that neurological disease related pathways including Huntington, Parkinson, and Alzheimer diseases were significantly inhibited by chronic ID (Figure 2B, Table S3).

### 2.5. Transcriptome Analysis of the Rat Hippocampus in Response to Iron Deficiency

**Figure 5:**
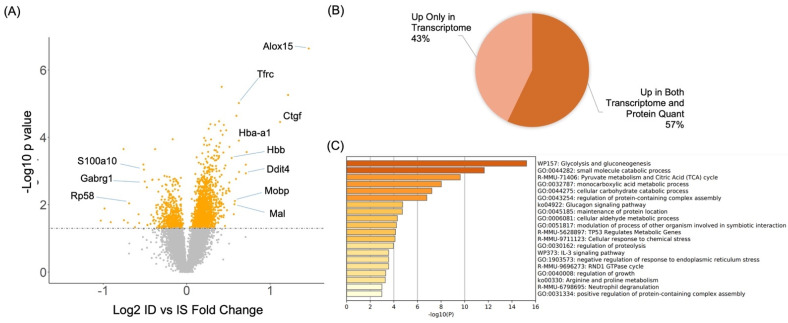
Gene expression analysis of ID-treated dynamics of transcriptome in postnatal day 15 rat hippocampus. (**A**) Volcano plot for transcriptome dynamic profiling of the rat hippocampus tissues. (**B**) Overlapping analysis between significantly upregulated genes in tissue with protein quantification data in cells. (**C**) Functional annotation enrichment of pathways and processes on proteins that were quantified to be upregulated based on both tissue and cell analysis.

To correlate the changes in proteome dynamics in iron-deficient HT22 cells with the gene expression changes in iron-deficient rat hippocampus, we compared the proteome to the hippocampal transcriptome of iron-deficient rats (P15), which identified 428 upregulated and 255 downregulated genes (*p* < 0.05, Figure 5A). Bioinformatics analysis of the upregulated genes showed that ID led to higher activities of diverse biological processes in rat hippocampal tissues including astrocyte differentiation, glycolysis, oxidative stress-induced neuron intrinsic apoptotic signaling, transition metal ion homeostasis, and response to hypoxia (Figure S1A). Cellular compartment analysis confirmed the neuronal specific effect with upregulated genes significantly enriched in myelin sheath and axon (Figure S1B). KEGG pathway analysis showed that 2-Oxocarboxylic acid metabolism, Glycolysis, and Biosynthesis of amino acids pathways were significantly enriched among upregulated genes in the iron-deficient rat hippocampus.

Among the differentially expressed genes from transcriptome profiling, 140 upregulated genes were also quantified in our SILAC-based quantitative proteomics analysis of HT22 cells with chronic iron deficiency treatment. a total of 80 of the 140 upregulated genes showed a higher protein abundance (57%) in our quantitative analysis of proteome dynamics in iron-deficient cells compared to iron-sufficient controls (Figure 5B), demonstrating a moderate correlation between protein dynamic in the iron-deficient HT22 neuronal cells and the transcriptome of the iron-deficient rat hippocampus. The Metascape-based functional enrichment analysis of the genes with an increased expression in both tissue and HT22 cells induced by ID showed enrichment in a number of biological processes including glycolysis, regulation of protein complex assembly, and cellular response to chemical stress (Figure 5C).

### 2.6. Transcriptional Factor Activity Enrichment Analysis in Iron-Deficient Rat Brain Tissue and Neuronal Cell Line

**Figure 6:**
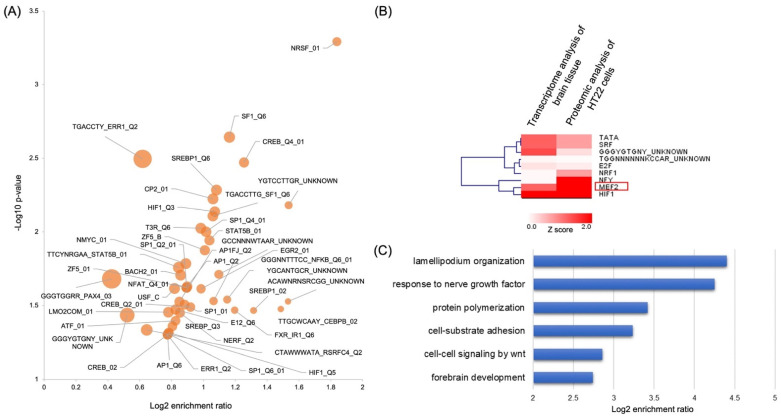
Transcription factor activity enrichment analysis. (**A**) Volcano plot of transcription factor enrichment analysis based on the transcriptome dynamic analysis in ID-induced P15 rat hippocampus. (**B**) Enrichment overlapping analysis of transcription factor activities showing a total of nine transcription factors that were activated by iron deficiency in both hippocampal tissue and HT22 cell line. (**C**) Pathway enrichment analysis of MEF2 targets quantified in the tissue transcriptome analysis.

The upregulated genes induced by iron-deficiency in the rat hippocampus were analyzed with a web-based gene set enrichment analysis (GSEA) [47] using the transcription factor target functional database. These in silico analyses uncovered 45 gene sets corresponding to 34 transcription factors (TFs) that were enriched by the upregulated genes (Figure 6A and Table S4). Nine of these TFs showed similar enrichment in both hippocampal transcriptome and HT22 cell proteome, including transcription factors that were known to associate with iron metabolism such as hypoxia-inducible factor 1 (HIF1), nuclear transcription factor Y (NFY), and nuclear respiratory factor 1 (NRF1) (Figure 6B). The activation of HIF1 in response to ID in both neuronal cells and tissues was expected as it is the master regulator of cellular metabolic sensing pathway upon microenvironmental stresses, such as hypoxia and iron deficiency [9,10,11]. NFY binds to the human ferritin promoter to activate the expression of ferritin, the major protein for iron storage in blood [48]. Upregulation of NFY activity upon chronic ID would promote intracellular iron storage as a potential compensatory mechanism. NRF1 activation is dependent on the production of reactive oxygen species (ROS) under hypoxia. Accordingly, ID might facilitate its activity to induce ROS production. NRF1 stimulates the expression of HO-1, ferritin, and the metallothionines 1 and 2 (MT1, MT2) [49,50], as well as iron sulfur-containing succinate dehydrogenase (SDH2) [51].

Among these TFs, myocyte enhancer factor 2 (MEF2) showed a strong enrichment in both hippocampal transcriptome and HT22 proteome. MEF2 is a transcription factor family that includes MEF2A, B, C, and D in human and involves in cell differentiation, migration, and metabolism [52]. To further understand the pathway regulated by MEF2 in brain tissue, we extracted MEF2-targeted genes in the transcriptome and performed pathway enrichment analysis. We found that these MEF2 downstream targets might involve in diverse neuronal developmental processes including lamellipodium organization, response to nerve growth factor, WNT signaling, and forebrain development (Figure 6C). These data demonstrated that iron deficiency in neuronal cells may regulate diverse signaling and developmental pathways in brain development through the activation of MEF2 transcription factor.

## 3. Discussion

Iron is an essential nutrient in mammalian cells and widely involved in diverse metabolic processes in neurons [31]. Iron level in tissues is maintained by dietary uptake of iron in the ferrous ion state and transported into the cell through divalent metal cation transporter 1 (DMT1) [53]. Under the regulation of hepcidin, iron is excreted into the plasma and transported via transferrin proteins into the neuronal cells. Iron deficiency is a nutritional stress that strongly affects the development of neurons. Previous studies by us and others have shown that early childhood iron deficiency, often due to malnutrition, led to profound neurological abnormalities in human that involve widespread changes in gene expression and epigenetics profiles, which cannot be reversed by subsequent iron supplementation [3,36,54,55]. System-wide discovery of early response cellular pathways and networks altered by iron deficiency in neurons will offer biochemical bases and potential therapeutic insights for the future development of translational strategies to mitigate the adverse physiological effects of neuronal iron deficiency.

ID has long been linked to oxygen deprivation due to the similar activation of HIF1-a pathways; yet, little is known on the similarities and differences between iron deficiency and hypoxia-induced changes in cellular proteome. Our recent global phosphoproteomics study revealed differential phosphorylation pathways caused by iron deficiency and hypoxia [21]. In this study, we performed global quantitative analysis of proteome to assess the comprehensive effects of hypoxia and acute and chronic iron deficiency on neuronal proteome dynamics. This study revealed early dynamics in cellular metabolism, signaling, and epigenetic regulation in response to these microenvironment challenges. The treatment conditions were designed so that HIF1-a was enriched at the same level under all conditions, thereby allowing our analyses to distinguish specifically the oxygen- and iron-dependent cellular pathways that were both dependent and independent of HIF1-a stabilization under each treatment.

Our pathway enrichment and clustering analysis showed some similarity between hypoxia and ID treatments in neuronal cells. In metabolism, both hypoxia and ID reduced the abundances of proteins involved in cholesterol metabolism and increased the abundances of proteins involved in fatty acid beta oxidation. In protein homeostasis, both hypoxia and ID significantly lowered the abundances of proteins involved in transcriptional activity, protein synthesis, and autophagy-mediated protein degradation pathways. In cellular signaling, both hypoxia and chronic iron deficiency led to the significant downregulation of mTORC1 signaling. As both hypoxia and ID led to the activation of HIF1-a protein through PHD-HIF1-a axis, potentially the similar effects between hypoxia and ID were mediated by the PHD-HIF1-a pathway. On the other hand, our analysis also revealed distinct biological processes that responded differently to hypoxia and iron deficiency. For example, in energy metabolism, chronic iron deficiency had a profound effect on classical hypoxia response pathways, including the upregulation of glycolysis pathways and the downregulation of electron transport chain in the oxidative phosphorylation pathway, while acute hypoxia treatment did not have a significant impact on the oxidative phosphorylation pathway, but had a stronger effect on the activation of fatty acid beta oxidation in neuronal cells. In protein homeostasis, only hypoxia treatment strongly affected the polyubiquitination pathway by reducing linkage-specific ubiquitination and upregulating the ERAD-mediated protein degradation pathway. In epigenetic regulation, iron deficiency and hypoxia regulated histone acetylation through a different mechanism. Iron deficiency (acute iron deficiency in particular) led to a decrease in the abundance of acetylation enzymes, including both histone deacetylases and acetyltransferases, while acute hypoxia significantly increased the abundances of members in histone acetyltransferase complexes, without strongly affecting the abundances of enzymes in the histone acetylation pathway. Hypoxia and chronic iron deficiency decreased the abundances of site-specific histone methylation pathways including H3K4, K9, K27, and K36, as well as DNA methylation. In iron ion regulation, both chronic and acute iron deficiency, but not hypoxia, led to the significant decrease in protein abundances involved in iron homeostasis and sequestration. Finally, in cell signaling, hypoxia, but not iron deficiency, significantly downregulated protein kinase B signaling, while iron deficiency, but not hypoxia, significantly upregulated the VEGF signaling pathway. Since all treatment conditions activated HIF1-a, the differential effects between hypoxia and ID treatments were likely driven by iron-dependent mechanisms unrelated to HIF1-a.

Mitochondria complex I is an essential component of the electron transport chain with eight iron–sulfur clusters. Reduced complex I activity in neurons is an underlying etiology for many neurological diseases, such as Parkinson disease and Alzheimer disease, and the normal function of complex I, is essential for neuronal development and neural stem cell differentiation [56,57,58]. The decreased complex I activity may also contribute to increased oxidative stress and protein damage in neurons [57]. Our CORUM complex enrichment analysis showed that chronic iron deficiency, but not acute iron deficiency or acute hypoxia, led to a significant downregulation of many members of mitochondrial complex I and thereby inhibited its activity. The ID-induced loss of complex I activity in neuron likely increases oxidative stress to proteins and lipids, compromising neuronal development.

The moderate congruency between HT22 proteome and P15 rat hippocampal transcriptome indicates a major effect of ID on neurons. The difference between these datasets was likely stemmed from non-neuronal effects of the whole hippocampal tissue. It is noteworthy that a common outcome of both datasets was the higher activity of HIF1, NFY, and NRF1, which are known to regulate cellular iron homeostasis [4,48,49,50,51]. Interestingly, we also identified MEF2 as a novel transcription factor induced by iron deficiency. While MEF2 is a well-known key transcription factor in myoblast differentiation and muscle formation [59], MEF2 activity can be also seen in specific excitatory synapses and play a critical role in neuronal development [60]. MEF2 activity is regulated by protein kinase A signaling pathway [61], which is hyperactivated in chronic iron deficiency [21]. Therefore, it is likely that iron deficiency in neurons modifies excitatory synaptic function by inducing MEF2 activity through protein kinase A signaling. Our study suggests a new iron deficiency-induced regulatory pathway that may contribute to the abnormal development in iron-deficient hippocampus.

Collectively, this study revealed distinct neuronal transcriptome and proteome responses to the hypoxia- or ID-induced stresses and confirmed that in vitro cell-based system can represent a good model to dissect the mechanisms by which iron availability alters gene regulation and protein dynamics in neuronal cells.

## 4. Materials and Methods

### 4.1. The P15 Rat Hippocampal Transcriptome

The P15 rat hippocampal transcriptome from our prior study [36] was re-analyzed using more advanced molecular tool kits in order to perform parallel analyses and to integrate with the proteomic data from HT22 neuronal cells. Total RNA from 15 rat hippocampi were processed for this study—iron deficient (*N* = 6) and iron sufficient (*N* = 9). Changes in gene expression between iron-deficient (experiment) and iron-sufficient (control) samples were determined using *p*-value < 0.05 adjusted for multiple comparison (Benjamini–Hochberg correction).

### 4.2. Quantitative Proteomics Analysis

#### 4.2.1. Cell Culture

HT22 mouse hippocampal neuronal cells (A gift from Dr. Schubert, Salk Institute, La Jolla, CA, USA) were maintained in Dulbecco’s modified Eagle’s cell culture medium (DMEM) (Gibco, Waltham, MA, USA) containing 1% penicillin–streptomycin (Corning, Glendale, AZ, USA) and 10% fetal bovine serum (FBS) (Sigma, St. Louis, MO, USA). Cells were incubated in a humidified cell culture incubator set at 37 °C and 5% CO_2_. To accomplish SILAC labeling, HT22 cells were cultured in DMEM for stable isotope labeling in cell culture (SILAC) (Thermo, Waltham, MA, USA). DMEM media for heavy SILAC labeling was supplemented with 10% dialyzed FBS (Gibco), 1% penicillin–streptomycin, 25 mg/500 mL proline, and ^13^C_6_^15^N_4_-l-arginine and ^13^C_6_^15^N_2_-l-lysine. DMEM media for light SILAC labeling was supplemented with 10% dialyzed FBS (Gibco), 1% penicillin–streptomycin, 25 mg/500 mL proline, and 50 mg/500 mL l-arginine and l-lysine. HT22 cells were labeled in SILAC media for over 6 generations before treatment. To study iron deficiency, deferoxamine (Sigma-Aldrich, St. Louis, MO, USA) was solubilized in DMSO. Heavy SILAC labeled HT22 cells were treated either for 6 h with 100 µM deferoxamine for acute iron deficiency or 24 h with 10 µM deferoxamine. Light SILAC labeled untreated control cells were prepared with a final DMSO vehicle concentration of 0.7%. To study hypoxia, heavy SILAC-labeled HT22 cells were incubated in a hypoxia chamber (1% O_2_, 94% N_2_, 5% CO_2_) set at 37 °C for 6 h. Light SILAC-labeled HT22 cells were prepared as a normoxia control by incubation in a humidified incubator (18% O_2_, 77% N_2_, 5% CO_2_) set at 37 °C for 6 h.

#### 4.2.2. Cell Lysis

Following treatment, HT22 cells were washed two times with cold PBS (Gibco, Life technologies). Cells were directly lysed on the plate with boiling lysis buffer composed of 6 M guanidinium hydrochloride (GnHCl), 100 mM Tris at pH 8.5, phosphatase inhibitor, and protease inhibitor. Cell lysate was collected, boiled for 10 min, solubilized by micro tip sonication, and processed by high-speed centrifugation for 10 min.

#### 4.2.3. Peptide Preparation

HT22 cell lysate protein concentration was measured by Bradford assay (Thermo, Waltham, MA, USA). The heavy and light SILAC labeled lysates were mixed together in a 1:1 (*w*/*w*) ratio. Proteins were reduced with tris(2-carboxyethyl)phosphine (TCEP) (5 mM), alkylated with iodoacetamide (5 mM), and blocked with cysteine (5 mM). Lysate was diluted to 1M GuHCl with 50 mM Tris, pH 8.5 and the pH was adjusted to pH 8.0 with 5 mM ammonium bicarbonate. Proteins were digested into peptides using Trypsin (Promega, Madison, WI, USA) using a 1:50 (*w*/*w*) enzyme/protein ratio overnight at 37 °C and again using a 1:100 (*w*/*w*) ratio for 2 h at 37 °C. The peptide sample was centrifuged for 10 min at 2000 rpm and concentrated with a Sep-Pak C18 cartridge (Waters, Milford, MA, USA). Peptides were extracted from the Sep-Pack cartridge using 1.2 mL 80% acetonitrile (ACN). The peptides were concentrated by vacuum centrifugation before storage at −80 °C.

#### 4.2.4. Offline High pH Reverse-Phase HPLC Fractionation

Up to 2 mg of peptides were solubilized in 10mM ammonia formate (pH 8.0). Peptides were fractionated using an Agilent 1100 HPLC (Agilent, Santa Clara, CA, USA). Peptides were loaded onto a Waters XBridge peptide BEH C18 column (3.5 μm, 4.6 × 150 mm) and separated using high pH reverse-phase chromatography. Buffer A was composed of 10 mM ammonia formate in water at pH 10.0 and buffer B was composed of 10 mM ammonia formate in 90% ACN at pH 10.0. Peptides were separated using a flow rate of 1 mL/min and a linear gradient of 3% B–35% B for 45 min, 35% B–95% B for 8 min, 95% B–3% B for 2 min and equilibrated at 3% B for 5 additional min. Fractions of 1 min were collected across the entire gradient and concatenated into a total of 4 fractions. Separated peptides were lyophilized and desalted with C18 spin columns (Thermo Scientific).

#### 4.2.5. LC-MS/MS Acquisition

Peptides were solubilized with 0.1% formic acid in water (*v/v*) and processed by an Orbitrap Fusion mass spectrometer (Thermo Scientific, Waltham, MA, USA) with an online Proxeon Easy nLC 1000 Nano-UPLC system (Thermo Scientific, Waltham, MA, USA). Peptides were processed on the Proxeon liquid chromatography system containing a self-packed nano column (50 cm × 100 µm, ReproSil-Pur Basic C18, 2.5 µm, Dr. Maisch GmbH) set at 55°C. Buffer A was composed of 0.1% formic acid in water (*v*/*v*) and buffer B was composed of 0.1% formic acid in acetonitrile (*v*/*v*). Peptides were separated using a flow rate of 300 nL/min and a gradient of 5% B–22% B for 79 min, 22% B–32% B for 11 min, 32% B–95% B for 10 min before column re-equilibration. Peptide ions were ionized by positive polarity electrospray and precursor ions were detected by the orbitrap. The Orbitrap acquisition parameters were set for a mass range of 380–1800 *m/z* and resolution of 120,000 at 200 *m/z*. Ions were then selected using a mass tolerance of ±25 ppm and using dynamic exclusion (15 s). Selected ions were fragmented with high energy collisional dissociation (HCD) set at 30%. Fragment ions were detected using the linear ion trap set with an isolation window of 1.6 *m/z*.

#### 4.2.6. Sequence Database Searching and Data Processing

Raw mass spectrometry data was processed with MaxQuant (version 1.5.3.12) [62]. The integrated Andromeda search engine performed peptide identification using default settings, the UniProt mouse database and a 1% false discovery rate (FDR) [63]. Additional settings included the fixed modification of cysteine carbamidomethylation and the variable modifications of methionine oxidation and protein N-termini acetylation. Two missed tryptic cleavages were permitted, and the quantification multiplicity was set at three. The median of the normalized peptide ratios was used to calculate the protein ratios. Only quantification from either unique peptides or peptides categorized as “Occam’s razor” were used for the median calculation.

### 4.3. Functional Annotation and Clustering

The protein quantification data was divided into four quantiles according to the normalized SILAC H/L ratio to perform clustering analysis. The quantiles were generated by dividing the data into four log2 ratio ranges: less than −1, −1 to 0, 0 to 1, and greater than 1, respectively. Statistical enrichment analysis using the hypergeometric test was performed for each quantile with the R package GOstats [64]. Enrichment analysis was performed using the Pfam domains, Kyoto Encyclopedia of Genes and Genomes (KEGG) pathway, and Gene Ontology—molecular functions, biological processes, and cellular compartment. The −log10 of the *p*-value enrichment outcome was calculated and normalized to calculate the z-score. For significance, 0.05 was set as the *p*-value cut-off threshold and 1.2 was set as the z-score cut-off threshold—data shared in Table S3. Each enrichment analysis category was processed with one-way hierarchical clustering (average linking and covariance value as the distance) of the functional annotation based on the z-score. CORUM protein complex enrichment analysis of chronic ID regulated proteins and functional annotation enrichment analysis of upregulated genes in Rat P15 hippocampus tissue (iron deficient/iron sufficient, *p* < 0.05) in P15 gene expression microarray was performed with WebGestalt focusing on Gene Ontology (Biological Processes, Cellular Compartment) and KEGG Pathway analysis with FDR cutoff of 0.05 [65]. Functional annotation enrichment analysis of overlapped genes of upregulation in both rat hippocampus tissue and chronic iron deficiency treatment in neuronal cells was performed in Metascape with p value cutoff of 0.05 [66].

### 4.4. Transcription Factor Enrichment

Significantly regulated proteins from the clustering analysis were analyzed with a Web-based Gene Set Enrichment Analysis (WebGestalt), using the transcription factor target functional database [65].

### 4.5. Western Blotting

To assess cellular treatment conditions, HT22 cells were grown to 80% confluency and incubated in a hypoxia chamber (1% O_2_, 94% N_2_, 5% CO_2_) set at 37 °C for 1.5, 3, 6, 12, 24 h. To study iron deficiency, HT22 cells were grown to 80% confluency and treated either for 6 h with 100 µM deferoxamine for acute iron deficiency, 24 h with 10 µM deferoxamine, and 6 h with DMSO as a vehicle control. These DFO treatments occurred in a humidified incubator (18% O_2_, 77% N_2_, 5% CO_2_) set at 37 °C. Cells were washed with PBS and lysed with Lysis buffer containing 150 mM NaCl, 1mM EDTA, 10 mM Tris-HCl pH 8.0, 0.1% Triton X-100 and protease inhibitor. For Western blotting, equal amounts of protein were loaded into each lane, separated by SDS-PAGE, transferred to PVDF membranes (Millipore, Burlington, MA, USA), before detection using anti actin (VWR, Radnor, Massachusetts) and anti-HIF1-a (Sigma) antibodies using the manufacturer’s instructions.

## Figures and Tables

**Table 1:** Dynamics of protein pathways regulated by hypoxia, acute iron deficiency, and chronic iron deficiency. Functional activity is summarized either as increased, decreased, or unchanged (blank).

CELLULAR FUNCTIONS	HYPOXIA	ACUTE ID	CHRONIC ID
TRANSCRIPTION	Decrease	Decrease	Decrease
PROTEIN SYNTHESIS	Decrease	Decrease	Decrease
MTORC1 SIGNALING	Decrease		Decrease
**PROTEIN DEGRADATION**			
UBIQUITIN MEDIATED	Decrease		
ENDOPLASMIC RETICULUM-ASSOCIATED	Increase		
AUTOPHAGY-MEDIATED	Decrease		Decrease
**EPIGENETIC PROCESSES**			
HISTONE DEACETYLASE		Decrease	
HISTONE ACETYLTRANSFERASE	Increase		Decrease
HISTONE METHYLATION	Decrease	Increase	Increase
**IRON ION REGULATION**			
IRON ION HOMEOSTASIS		Decrease	Decrease
**CELLULAR METABOLISM**			
CHOLESTEROL BIOSYNTHESIS	Decrease	Decrease	Decrease
FATTY ACID METABOLISM	Decrease		
BETA OXIDATION	Increase		
GLYCOLYSIS		Increase	Increase
ELECTRON-TRANSPORT CHAIN			Decrease
PROTEIN KINASE B SIGNALING	Decrease		
VEGF SIGNALING		Increase	Increase
GLUTAMATE SECRETION			Increase

## Data Availability

The data presented in this study are openly available at the ProteomeXchange Consortium open database repository with identifier PXD030380.

## Supplementary Materials

The following supporting information can be downloaded. Figure S1: Functional annotation enrichment analysis of genes that significantly upregulated in Rat hippocampus tissue; Table S1: Hypoxia and DFO proteinGroups Quantitation, Identification and quantification of proteins in HT22 cells upon acute hypoxia treatment (1% oxygen for 6 h), acute iron deficiency treatment (100 µM DFO for 6 h), and chronic iron deficiency treatment (10 µM DFO for 24 h), Table S2: HIF1a targets quantitation in hypoxia and DFO, The list of known transcriptional targets of hypoxia-inducible factor 1 alpha and corresponding quantification upon hypoxia, acute and chronic iron deficiency treatment, Table S3: Gene Ontology Annotation for (A) Biological Process, (B) KEGG Pathways, (C) Molecular Functions, (D) PFAM Analysis, Table S4: P15 microarray raw data, Quantification of gene expression profiles with microarray analysis on P15 rat hippocampus tissue upon iron deficiency diet.
