# Supplementary material for: Quantitative Proteome and Transcriptome Dynamics Analysis Reveals Iron Deficiency Response Networks and Signature in Neuronal Cells
Source: Molecules. 2022 Jan 13;27(2):484. doi: 10.3390/molecules27020484 (PMC8779535; doi:10.3390/molecules27020484)
Supplement: Supplementary file 1 [file molecules-27-00484-s001.zip › ID and Hypoxia prot quant - Supp Info-Final.pdf]

Quantitative Proteome and Transcriptome Dynamics Analysis Reveals Iron Deficiency Response Networks and Signature in Neuronal Cells

Luke Erber [1], Shirelle Liu [2], Yao Gong [1], Phu Tran [2,*] and Yue Chen [1,*]

[1] Department of Biochemistry, Molecular Biology and Biophysics, University of Minnesota at Twin Cities, Minneapolis, MN 55455, USA; lerber@umn.edu (L.E.) gong0062@umn.edu (Y.G.)
[2] Department of Pediatrics, University of Minnesota at Twin Cities, Minneapolis, MN 55455, USA; liu00459@umn.edu (S.L.)
* Correspondence: tranx271@umn.edu (P.T.); yuechen@umn.edu (Y.C.)

Supplementary Information

Supplemental Tables

Table S1. Hypoxia and DFO treatment protein groups quantitation. Identification and quantification of proteins in HT22 cells upon acute hypoxia treatment (1% oxygen for 6 hours), acute iron deficiency treatment (100 µM DFO for 6 hours), and chronic iron deficiency treatment (10 µM DFO for 24 hours).

Table S2. HIF1a targets quantitation in hypoxia and DFO treatments. The list of known transcriptional targets of Hypoxia-inducible Factor 1 alpha and corresponding quantification upon hypoxia, acute and chronic iron deficiency treatment.

Table S3. Gene Ontology Annotation for A) Biological Process, B) KEGG Pathways, C) Molecular Functions, D) PFAM Analysis.

Table S4. P15 microarray raw data. Quantification of gene expression profiles with microarray analysis on P15 rat hippocampus tissue upon iron deficiency diet.

Supplemental Figure

Figure S1. Functional annotation enrichment analysis of genes that significantly upregulated in Rat hippocampus tissue in response to iron deficiency ($p<0.05$) using WebGestalt focusing on (A) Gene Ontology Biological Processes, (B) Cellular Compartment and (C) KEGG Pathway.

(A)

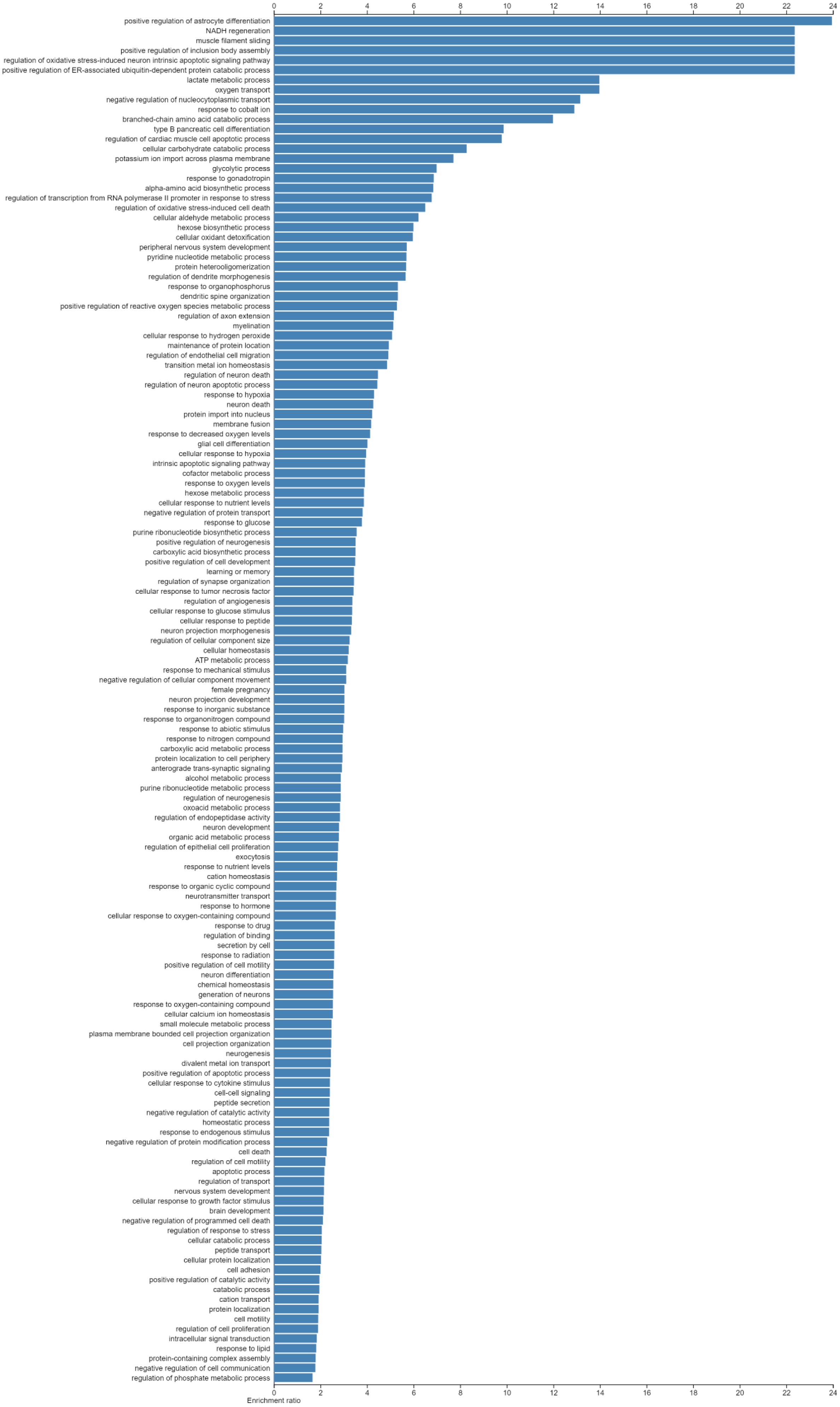
0 2 4 6 8 10 12 14 16 18 20 22 24
positive regulation of astrocyte differentiation
NADH regeneration
muscle filament sliding
positive regulation of inclusion body assembly
regulation of oxidative stress-induced neuron intrinsic apoptotic signaling pathway
positive regulation of ER-associated ubiquitin-dependent protein catabolic process
lactate metabolic process
oxygen transport
negative regulation of nucleocytoplasmic transport
response to cobalt ion
branched-chain amino acid catabolic process
type B pancreatic cell differentiation
regulation of cardiac muscle cell apoptotic process
cellular carbohydrate catabolic process
potassium ion import across plasma membrane
glycolytic process
response to gonadotropin
alpha-amino acid biosynthetic process
regulation of transcription from RNA polymerase II promoter in response to stress
regulation of oxidative stress-induced cell death
cellular aldehyde metabolic process
hexose biosynthetic process
cellular oxidant detoxification
peripheral nervous system development
pyridine nucleotide metabolic process
protein heterooligomerization
regulation of dendrite morphogenesis
response to organophosphorus
dendritic spine organization
positive regulation of reactive oxygen species metabolic process
regulation of axon extension
myelination
cellular response to hydrogen peroxide
maintenance of protein location
regulation of endothelial cell migration
transition metal ion homeostasis
regulation of neuron death
regulation of neuron apoptotic process
response to hypoxia
neuron death
protein import into nucleus
membrane fusion
response to decreased oxygen levels
glial cell differentiation
cellular response to hypoxia
intrinsic apoptotic signaling pathway
cofactor metabolic process
response to oxygen levels
hexose metabolic process
cellular response to nutrient levels
negative regulation of protein transport
response to glucose
purine ribonucleotide biosynthetic process
positive regulation of neurogenesis
carboxylic acid biosynthetic process
positive regulation of cell development
learning or memory
regulation of synapse organization
cellular response to tumor necrosis factor
regulation of angiogenesis
cellular response to glucose stimulus
cellular response to peptide
neuron projection morphogenesis
regulation of cellular component size
cellular homeostasis
ATP metabolic process
response to mechanical stimulus
negative regulation of cellular component movement
female pregnancy
neuron projection development
response to inorganic substance
response to organonitrogen compound
response to abiotic stimulus
response to nitrogen compound
carboxylic acid metabolic process
protein localization to cell periphery
anterograde trans-synaptic signaling
alcohol metabolic process
purine ribonucleotide metabolic process
regulation of neurogenesis
oxoacid metabolic process
regulation of endopeptidase activity
neuron development
organic acid metabolic process
regulation of epithelial cell proliferation
exocytosis
response to nutrient levels
cation homeostasis
response to organic cyclic compound
neurotransmitter transport
response to hormone
cellular response to oxygen-containing compound
response to drug
regulation of binding
secretion by cell
response to radiation
positive regulation of cell motility
neuron differentiation
chemical homeostasis
generation of neurons
response to oxygen-containing compound
cellular calcium ion homeostasis
small molecule metabolic process
plasma membrane bounded cell projection organization
cell projection organization
neurogenesis
divalent metal ion transport
positive regulation of apoptotic process
cellular response to cytokine stimulus
cell-cell signaling
peptide secretion
negative regulation of catalytic activity
homeostatic process
response to endogenous stimulus
negative regulation of protein modification process
cell death
regulation of cell motility
apoptotic process
regulation of transport
nervous system development
cellular response to growth factor stimulus
brain development
negative regulation of programmed cell death
regulation of response to stress
cellular catabolic process
peptide transport
cellular protein localization
cell adhesion
positive regulation of catalytic activity
catabolic process
cation transport
protein localization
cell motility
regulation of cell proliferation
intracellular signal transduction
response to lipid
protein-containing complex assembly
negative regulation of cell communication
regulation of phosphate metabolic process
0 2 4 6 8 10 12 14 16 18 20 22 24
Enrichment ratio

(B)

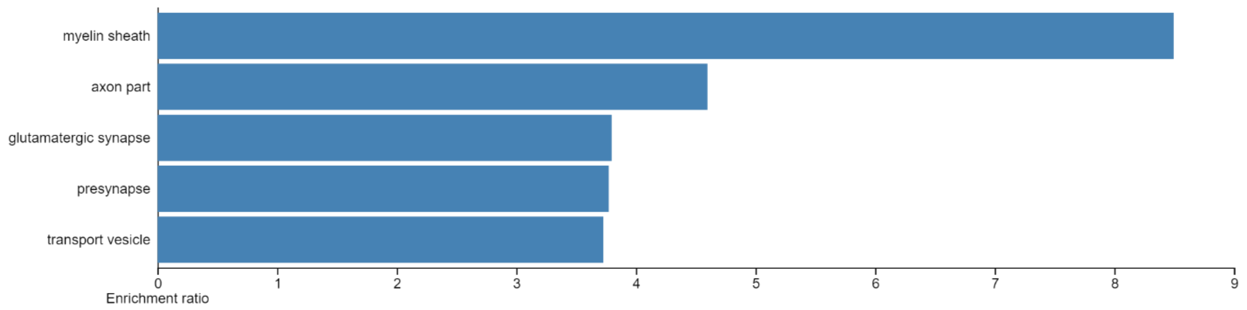
myelin sheath
axon part
glutamatergic synapse
presynapse
transport vesicle
0
1
2
3
4
5
6
7
8
9
Enrichment ratio

(C)

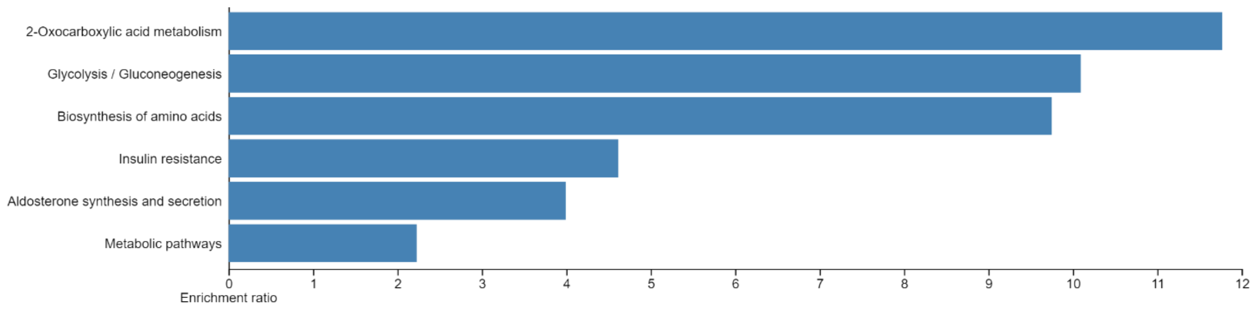
2-Oxocarboxylic acid metabolism
Glycolysis / Gluconeogenesis
Biosynthesis of amino acids
Insulin resistance
Aldosterone synthesis and secretion
Metabolic pathways
0
1
2
3
4
5
6
7
8
9
10
11
12
Enrichment ratio
